# Supplementary material for: A systematic review of evidence for the added benefits to health of exposure to natural environments
Source: BMC Public Health. 2010 Aug 4;10:456. doi: 10.1186/1471-2458-10-456 (PMC2924288; doi:10.1186/1471-2458-10-456)
Supplement: Additional file 1 — Details of search strategy. [file 1471-2458-10-456-S1.DOC]

**Search strategy**

*Databases/Library:*

PubMed; EMBASE; CINAHL; PsycINFO; Web of Science (Science and Social science); SPORT Discus; ASSIA; HMIC- Data ; LILACS; UK National Research Register archives; TRIP database; UK National Library for Health ; Index to Theses Online; Directory of Open Access Journals; Economic and Social Data Service; Database of Promoting Health Effectiveness Reviews; Trials Register of Promoting Health Interventions; Cochrane Collaboration and Campbell Collaboration.

*Websites searched:*

British Heart Foundation (incl. National Centre for Physical Activity and Health); British Trust for Conservation Volunteers; Canadian Health Network; Commission for Architecture and the Built Environment; Centre for Child and Family Research; Countryside Recreation Network; Countryside Council for Wales; Department of Health ; Environment agency; EU Cost Action E39 ; European Environment Agency; Forest Research; Forestry Commission; Glasgow Centre for Public Health; Greenspace (including Greenspace Scotland); Groundwork; Health Development Agency; Health Protection Agency; Health Technology Assessment; Institute of Rural Health; Living Streets; Mental Health Foundation; Mind; Medical Research Council; National Library for Public Health; National Public Health Service for Wales; National Parks; National Trust; Natural England; The new economics foundation; NICE; OPENspace; Parks Victoria; Royal Society for the Protection of Birds; Scottish Executive; Sustainable Development Commission; Sustainable Development Research Network; Scottish Environment Protection Agency; Scottish Natural Heritage; SNIFFER; Sustrans; THRIVE; The Nature Conservancy; The Woodland Trust; UK MAB Urban Forum; UK National Research Register Archive; UK Public Health Association; World Health Organisation; Wildlife Trusts; World Wide Fund for Nature

### Search terms:

Combinations of the following search terms (where * denotes a wild card term) were used to search the databases and internet web sites. When appropriate, relevant Medical Subject Headings were also searched for within a database and also added to the search term list.

*Health terms*: Exerc*, Physical activit*, Walk*, Health, Restorati*, Recovery, Therap*, Well-being, Wellbeing, Well being, Pyscholog*, Quality of Life, Life satisfaction, Play, Social, Recreation, Self*, Personal development, Child development, Happiness, Morale, Anxiety, Depression, Stress, Pain

*Environmental terms*: Park, Parks, Green*, Natural environment*, Open space*, Garden*, Horticultur*, Wilderness, Countryside, Outdoors, Nature, Biodiversity, Wood*, Allotment*, Forest*
