# Supplementary material for: A systematic review of evidence for the added benefits to health of exposure to natural environments
Source: BMC Public Health. 2010 Aug 4;10:456. doi: 10.1186/1471-2458-10-456 (PMC2924288; doi:10.1186/1471-2458-10-456)
Supplement: Additional file 2 — References of articles included in the review and summaries of their basic characteristics. [file 1471-2458-10-456-S2.DOC]

References of articles included in the review and summaries of their basic characteristics.

| **Citation** | **Activity/Comparator** | **Participants** | **Methodology** | **Main outcomes category** |
| --- | --- | --- | --- | --- |
| Berman et al. (2008)  [41] | Walk in a park vs downtown urban area for 50-55min | 38 students  (61% female; mean age=22.62 yrs old) | Pretest-posttest crossover trial  (order randomised/balanced) | Emotions, Attention test |
| Bodin & Hartig (2003)  [42] | Run through a large nature reserve vs an urban area for 60 mins | 12 regular runners  (50% female; 26-46 yrs old) | Pretest-posttest crossover trial  (order randomised/balanced) | Emotions, Attention test |
| Butryn & Furst (2003)  [43] | Run through a public park vs an urban area along a 4 mile course | 30 non-elite distance runners  (100% female; 18-55 yrs old) | Pretest-posttest crossover trial  (order counterbalanced) | Emotions |
| Cuvo et al. (2001)  [27] | Activities in a living/Snoezelen rooms vs in outdoor grounds over 13 sessions | 3 adults with profound mental retardation (33% female; 44-65 yrs old) | Crossover trial: assessments during activity (counterbalanced) | Idiosyncratic symptoms e.g. body rocking. Engagement |
| Faber Taylor et al. (2001)  [48] | Afterschool and weekend activities in indoor, built vs green settings | 96 parents of children diagnosed with ADD/ADHD (25% female; 7-12 yrs) | Observational study: questionnaire completed by parents | Attention (ADHD symptoms as rated by their parents) |
| Faber Taylor & Kuo (2009) [34] | Walk in a park vs an urban area for 20 mins (‘downtown’ or ‘neighbourhood’) | 17 children diagnosed with ADHD  (12% female; 7-12 yrs old) | Posttest crossover trial  (order randomised/balanced) | Attention test |
| Harte & Eifert (1995)  [45] | Run on a university campus vs treadmill (with different stimuli) for 45 mins^1^ | 10 amateur triathletes or marathon runners (100% male; 18-37 yrs old) | Pretest-posttest crossover trial (order counterbalanced) | Emotions, Endocrine,  Blood pressure |
| Hartig et al. (1991)  Study 1 [32a] | Wilderness backpacking trip vs non-wilderness vacation for 4-7 days^1^ | 68 physically-fit backpackers  (38% female; mean age=32 yrs old) | Observational study: before and after assessments | Emotions, Attention test |
| Hartig et al. (1991)  Study 2 [32b] | Walk in a park vs an urban area for 40 mins^1^ | 102 college students  (50% female) | Pretest-posttest comparison groups (randomised) | Emotions, Attention test, Cardiovascular |
| Hartig et al. (1999)  [47] | Being in a garden vs urban area for c.15 mins (words or personal memories test) | 101 students  (61% female; mean age=20.6 yrs old) | Posttest comparison groups  (randomised) | Emotions, Memory recall |
| Hartig et al. (2003)  [35] | Walk in a wildlife preserve vs urban area for 50 mins (with or without pretrial task) | 112 students screened for health factors (50% female; mean age=20.8 yrs old) | Pretest-posttest comparison groups (block**-**randomised)DURING | Emotions, Attention test,  Blood pressure |
| Hull & Michael (1995)  [46] | Passive leisure activities in a suburban park vs at home for 45 min to 3 hr | 20 - mostly college students  (50% female) | Observational study: questionnaire  completed before, during and after | Emotions |
| Isaacs et al. (2007)  [28] | Walk in parks/open space vs leisure-centre based exercise 10-wk programs^1^ | 943 adults not physically active with CVD risk (67.3% female; 40 - 74 yrs) | Pretest-posttest comparison groups  (block randomised) | Various incl. Emotions, Cardiovascular, Fitness |
| Kerr et al. (2006)  [44] | Run on a university footpath vs laboratory treadmill for 5km | 22 recreational and 22 competitive runners (100% male; mean age=21) | Pretest-posttest crossover trial  (order counter-balanced) | Emotions |
| Kuo & Faber Taylor (2004)  [49] | Afterschool and weekend activities in indoor, built vs green settings | 452 parents of children diagnosed with ADHD (21% female; 5 -18 yrs old) | Observational study: questionnaire completed by parents | Attention (ADHD symptoms as rated by their parents) |
| Li et al. (2008)  [36] | Walk during a 3 day trip to forest vs 3 day trip to city | 12 “healthy” adults  (100% male; 35-56 yrs old) | Pretest-posttest crossover trial  (same order) | Immune function, Endocrine, Sleeping hours |
| Park et al. (2007)  [30] | Walk/being within a forest vs in a city for 20 mins | 12 students with no health disorders (100% male; mean age=22.8 yrs old) | Pretest-posttest crossover trial  (order randomised/balanced) | Emotions, Endocrine, Cerebral activity |
| Peacock et al. (2007)  [33] | Walk around a country park vs indoor shopping centre for 1h | 20 members of local “Mind” groups  (65% female; 31-70 yrs old) | Pretest-posttest crossover trial  (same order) | Emotions |
| Plante et al. (2006)  [37] | Walk on a college campus vs treadmill watching a video of walk for 20 mins^1^ | 112 university students  (58% female) | Pretest-posttest comparison groups (randomised) | Emotions |
| Plante et al. (2007)  [38] | Walk on a college campus vs treadmill for 20 mins (alone or with friend) | 88 university students  (100% female; 18-22 yrs old) | Pretest-posttest comparison groups (randomised) | Emotions |
| Scholz & Krombholz (2007) [29] | Attendance of a forest kindergarten vs a regular kindergarten | 129 children with no physical problems (45% female; aged at least 60 mths) | Observational study: assessments made using developed tests | Motor performance ability |
| Teas et al. (2007)  [39] | Walk on a university path vs on a treadmill in a gym for 1 hr | 19 active postmenopausal women  (mean age=58 yrs old) | Pretest-posttest crossover trial  (same order) | Emotions, Endocrine, Blood pressure |
| Telles & Srinivas (1998)  [26] | Physical activity in the garden (gardening) vs yoga for 3 wks | 24 children with impaired vision aged (12-17 yrs old) | Pretest-posttest comparison groups  (randomised) | Cardiovascular |
| Tsunetsugu et al. (2007)  [31] | Walk/being within a forest vs in a city for 15 mins | 12 university students  (100%male; 21-23 yrs old) | Pretest-posttest crossover trial  (order randomised/balanced) | Emotions, Cardiovascular, Endocrine, Immune function |
| Yamaguchi et al. (2006)  [40] | Walk/being within a forest vs in a city for 20 mins | 10 university students  (100% male) | Pretestpost test crossover trial  (order balanced) | Endocrine |

^1^These studies also contained additional comparisons not presented in this table; in most cases, the additional comparison was an inactive or no treatment group.
