# Supplementary material for: A systematic review of evidence for the added benefits to health of exposure to natural environments
Source: BMC Public Health. 2010 Aug 4;10:456. doi: 10.1186/1471-2458-10-456 (PMC2924288; doi:10.1186/1471-2458-10-456)
Supplement: Additional file 3 — Effect size (and standard error of the effect size) calculated from each article for the most commonly reported outcomes. [file 1471-2458-10-456-S3.DOC]

## Effect size (and standard error of the effect size) calculated from each article for the most commonly reported outcomes (data from at least four studies available). The sign of the effect size reflects the benefit to health (positive effects indicate greater attention, energy and tranquillity but lower values for the other outcomes). Subgroups refer to additional factors investigated within a study.

| **Article citation** | **Subgroup** | **Tests of attention** | **Energy/**  **vigour** | **Anxiety/**  **tension** | **Calmness/ tranquility** | **Anger/**  **aggression** | **Fatigue/**  **tiredness** | **Sadness/**  **depression** | **Systolic**  **BP** | **Diastolic BP** | **Cortisol Conc.** |
| --- | --- | --- | --- | --- | --- | --- | --- | --- | --- | --- | --- |
| Berman et al. (2008) | NA | 0.44 *(0.24)* |  |  |  |  |  |  |  |  |  |
| Bodin & Hartig (2003) | females | -0.1 *(0.58)* | 0.15 *(0.58)* | - | 0.26 *(0.58)* | -0.1 *(0.58)* | - | - | - | - | - |
|  | Males | -0.08*(0.58)* | 0.19 *(0.58)* | - | 0.22 *(0.58)* | 1.13 *(0.64)* | - | - | - | - | - |
| Butryn & Furst (2003) | NA | - | 0.08 *(0.25)* | - | 0.41 *(0.26)* | - | - | - | - | - | - |
| Faber Taylor & Kuo (2009) | downtown | 0.51 *(0.35)* | - | - | - | - | - | - | - | - | - |
|  | neighbourhood | 0.57 *(0.35)* | - | - | - | - | - | - | - | - | - |
| Harte and Eifert (1995) | external stimuli | - | - | -0.09*(0.45)* | - | -0.61*(0.46)* | 0.31 *(0.45)* | 1.19 *(0.49)* | 0.26 *(0.45)* | - | 0.1 *(0.45)* |
|  | internal stimuli | - | - | 0.54 *(0.46)* | - | 1.62 *(0.53)* | 1.21 *(0.50)* | 1.09 *(0.49)* | 0.20 (0.45) | - | 0.32 *(0.45)* |
| Hartig et al. (1991; study 2) | NA | 0.47 *(0.26)* | - | - | - | 0.42 *(0.26)* | - | 0.31 *(0.25)* | -0.12*(0.25)* | -0.27*(0.26)* | - |
| Hartig et al. (1999) | word recall |  |  |  |  | 0.49 *(0.29)* |  | 0.1 *(0.29)* |  |  |  |
|  | personal memories |  |  |  |  | 0.37 *(0.28)* |  | 0.36 *(0.28)* |  |  |  |
| Hartig et al. (2003) | no task | -0.16*(0.28)* | - | - | - | 1.03 *(0.29)* | - | - | 0.26 *(0.29)* | 0.14 *(0.29)* | - |
|  | pre-treatment task | 0.06 *(0.28)* | - | - | - | 0.13 *(0.28)* | - | - | 0 *(0.28)* | 0.35 *(0.29)* | - |
| Kerr et al. (2006) | competitive runners | - | - | 0 *(0.30)* | 0.34 *(0.30)* | 0.41 *(0.30)* | - | - | - | - | - |
|  | recreational runners | - | - | 0.26 *(0.30)* | -0.25*(0.30)* | 0.38 *(0.30)* | - | - | - | - | - |
| Park et al. (2007) | NA | - | - | - | 1.34 *(0.46)* | - | - | - | - | - | -0.4 *(0.41)* |
| Peacock et al. (2007) | NA | - | 0.36 *(0.34)* | 0.54 *(0.35)* | - | 0.38 *(0.34)* | 0.33 *(0.34)* | 0.41 *(0.34)* | - | - | - |
| Plante et al. (2006) | females | - | 1.1 *(0.33)* | -1.29*(0.33)* | -0.87*(0.32)* | - | 1.02 *(0.32)* | - | - | - | - |
|  | Males | - | 0.08 *(0.35)* | -0.75*(0.37)* | -0.11*(0.35)* | - | -0.38*(0.36)* | - | - | - | - |
| Plante et al. (2007) | Alone | - | 0.07 *(0.30)* | 0.47 *(0.31)* | -0.08*(0.30)* | - | 0.7 *(0.31)* | - | - | - | - |
|  | with a friend | - | 0.5 *(0.31)* | 0.09 *(0.30)* | 0.42 *(0.31)* | - | 0.14 *(0.30)* | - | - | - | - |
| Teas et al. (2007) | NA | - | - | 0.56 *(0.33)* | - | 0.52 *(0.33)* | - | 0.21 *(0.33)* | 0.12 *(0.32)* | 0.29 *(0.33)* | -0.35*(0.33)* |
| Tsunetsugu et al. (2007) | NA | - | - | - | 1.65 *(0.57)* | - | - | - | 0.25 *(0.47)* | 0.29 *(0.47)* | 0.87 *(0.47)* |
