# Supplementary material for: A systematic review of evidence for the added benefits to health of exposure to natural environments
Source: BMC Public Health. 2010 Aug 4;10:456. doi: 10.1186/1471-2458-10-456 (PMC2924288; doi:10.1186/1471-2458-10-456)
Supplement: Additional file 4 — Adjusted effect sizes (posttest - pretest effect size) and 95% CI to investigate sensitivity of results to any pretest differences. [file 1471-2458-10-456-S4.DOC]

**Analysis to investigate sensitivity of results to any pretest differences:**

**Effect sizes (Hedges g) and 95 % CI of adjusted posttest effect sizes**

**(adjusted = posttest effect size minus pretest effect size when pretest data were available)**

The sign of the effects reflect the benefit on health (positive effects indicate greater attention, energy and tranquillity but lower values for the other outcomes). The number of studies is the number of studies used to calculate the effect (in brackets is the number of studies with pretest data). An asterisk is used to denote a significant heterogeneity test (p<0.05) for a particular group.

| Outcome | Effect  size | 95 % CI | No. studies | Summary |
| --- | --- | --- | --- | --- |
| Attention | 0.240 | (-0.120, 0.600) | 5 (3) | No effect |
| Energy | 0.145 | (-0.142, 0.433) | 5 (5) | No effect |
| Anxiety | 0.378 | (-0.065, 0.821) | 6 (6) | No effect* |
| Tranquillity | 0.446 | (-0.096, 0.987) | 7 (7) | No effect* |
| Anger | 0.568 | (0.343, 0.794) | 8 (6) | Improved |
| Fatigue | 0.684 | (-0.098, 1.466) | 4 (4) | No effect* |
| Sadness | 0.383 | (0.106, 0.660) | 5 (3) | Improved |
| Systolic BP | 0.080 | (-0.207, 0.368) | 5 (4) | No effect |
| Diastolic BP | 0.034 | (-0.271, 0.339) | 4 (3) | No effect |
| Cortisol conc. | -0.437 | (-1.671, 0.798) | 4 (4) | No effect* |
